# Machine Learning‐Based Identification of Children With Intermittent Exotropia Using Multiple Resting‐State Functional Magnetic Resonance Imaging Features

**DOI:** 10.1002/brb3.70556

**Published:** 2025-05-13

**Authors:** Mengdi Zhou, Huixin Li, Xiaoxia Qu, Lirong Zhang, Xueying He, Xiwen Wang, Jie Hong, Jing Fu, Zhaohui Liu

**Affiliations:** ^1^ Department of Radiology, Beijing Tongren Hospital Capital Medical University Beijing China; ^2^ Beijing Tongren Eye Center, Beijing Tongren Hospital, Capital Medical University, Beijing Key Laboratory of Ophthalmology & Visual Sciences Beijing China; ^3^ Department of Radiology Aerospace Center Hospital Beijing Beijing China; ^4^ Department of Radiology The Affiliated Drum Tower Hospital of Nanjing University Medical School Nanjing Jiangsu China

**Keywords:** Intermittent exotropia, machine learning, resting‐state functional magnetic resonance imaging, spontaneous activity

## Abstract

**Objective:**

To investigate the performance of machine learning (ML) methods based on resting‐state functional magnetic resonance imaging (rs‐fMRI) parameters in distinguishing children with intermittent exotropia (IXT) from healthy controls (HCs).

**Method:**

Forty‐one IXT children and 36 HCs were recruited. The amplitude of low‐frequency fluctuations (ALFF), fractional ALFF (fALFF) in the slow‐4 and slow‐5 bands, and regional homogeneity (ReHo) were calculated. The 360 cortical areas of the Human Connectome Project multimodal parcellation atlas (HCP‐MMP 1.0 atlas) were chosen as 360 regions of interest (ROIs). Each rs‐fMRI parameter value of one ROI was taken as a feature. The Pearson correlation coefficient (PCC) was performed to reduce dimensions. We used four feature selection methods and nine classifiers. The ten‐fold cross‐validation was applied to evaluate the results.

**Results:**

The ML methods combined with rs‐fMRI parameters had good classification performance in distinguishing IXT children from HCs, with the slow‐5 fALFF parameter showing the best classification performance. The linear regression (LR) classifier with analysis of variance (ANOVA) feature selection achieved the highest area under the receiver operator characteristic curve values (0.957, 0.804, and 0.818 for the training, validation, and test datasets, respectively) using five features, including the slow‐5 fALFF values of the right inferior parietal gyrus (IPG), right supplementary motor area (SMA), left primary somatosensory complex, right frontal opercula, and left dorsolateral prefrontal cortex (DLPFC), and the accuracy, sensitivity, and specificity values were 0.759, 0.759, and 0.760, respectively. The brain regions showing the greatest discriminative power included right IPG, right SMA, left primary somatosensory complex, right frontal opercula, left DLPFC, right posterior orbitofrontal cortex (pOFC), left medial superior temporal (MST), left parieto‐occipital sulcus (POS), and right anterior ventral insula.

**Conclusion:**

Based on the slow‐5 fALFF values of the five cortices as the features, LR with ANOVA was the best ML model for distinguishing between IXT children and HCs. The result indicates the slow‐5 fALFF parameter has the potential to serve as a biomarker for distinguishing IXT children from HCs. In addition, brain regions related to stereopsis, eye movement, and higher‐order cognitive functions play an important role in the neuropathologic mechanisms underlying IXT.

## Graphical abstract

The machine learning methods combined with resting‐state functional magnetic resonance imaging parameters (ALFF, slow‐5 fALFF, and ReHo) had good classification performance in distinguishing intermittent exotropia (IXT) children from healthy controls (HCs). Among several parameters, the slow‐5 fALFF showed the best classification performance. The slow‐5 fALFF can be a promising biomarker for distinguishing IXT children from HCs.

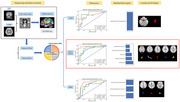

## 1 Introduction

Intermittent exotropia (IXT) is the most prevalent type of strabismus in childhood, with an incidence rate of approximately 1% in the United States and up to 4% in Asia (Fu et al. 2014; Govindan et al. 2005; McKean‐Cowdin et al. 2013; Yu et al., 2002). It is a disorder of ocular alignment characterized by an intermittent outward deviation of the eye, especially when the patient is inattentive, tired, or looking at a distance (Cotter et al. 2020). Cosmetic changes caused by eye deviation make children suffer from exclusion and prejudice, significantly increasing the risk of mental disorders in children. Although IXT is usually diagnosed through clinical examination (Sprunger et al. 2023), missed diagnosis frequently occurs because of the intermittent nature of IXT and the limited competency of children. During critical periods in the development of visual functions, the visual development of IXT children is plastic (Harwerth et al. 1986; Kiorpes 2015). If left untreated, childhood‐onset IXT is likely to deteriorate over time, potentially progressing to constant exotropia (Zhao et al. 2021). Therefore, early diagnosis and optimal treatment are crucial in the management of children with IXT.

Although numerous studies based on functional magnetic resonance imaging (fMRI) proposed strabismus is an abnormality of the central nervous system, different types of strabismus have individualized mechanisms and characteristic brain changes. Three literatures have reported brain alterations of IXT children, which contributed to comprehending the underlying neuropathologic mechanisms. Firstly, fusion function and vergence eye movement‐associated brain regions were investigated in IXT children by task‐fMRI (Zhang et al. 2023). However, in contrast to resting‐state fMRI (rs‐fMRI), task‐fMRI cannot avoid potential performance confounders associated with activation tasks (Tan et al. 2016; Yang et al. 2014). Using rs‐fMRI, IXT children were found to have abnormal changes in amplitude of low‐frequency fluctuations (ALFF), fractional ALFF (fALFF), and functional connectivity (FC) in brain areas related to vision and eye movement, such as reduced ALFF in the bilateral calcarine sulcus and cuneus, reduced fALFF in the left lingual gyrus and bilateral inferior parietal lobule (IPL), reduced FC between the left frontal eye field and bilateral IPL, and so on . (Fei et al. 2023; Zhu et al. 2023). Nevertheless, whether these brain regions have diagnostic value is unknown. Brain abnormalities of IXT adults observed in previous studies could not accurately reflect brain changes of childhood‐onset IXT (He et al. 2021; He et al. 2021; Li et al. 2024; Xia et al. 2024; Yang et al. 2014). Additionally, although there are many studies on the brain alterations of patients with comitant strabismus (CS) and strabismus including many subtypes (Chen et al. 2021; Liu et al. 2022; Shao et al. 2019; Tan et al. 2016; Zhu et al. 2018), these studies cannot reflect the occurrence and progression mechanism underlying IXT. Previous studies on IXT predominantly utilized univariate analysis to identify localizing alterations based on group‐level differences, ignoring the information in spatial distribution patterns. Thus, whether group differences can be useful for informing the diagnosis of individual IXT children remains unclear.

To characterize the fluctuating patterns of brain activity, various rs‐fMRI indices have been proposed. Among these, ALFF/fALFF (Fei et al. 2023; He et al. 2021; Zhu et al. 2023; Zou et al. 2008) and regional homogeneity (ReHo) (Zang et al. 2004) are the most commonly utilized functional indices in fMRI studies. ALFF is a reliable metric that reflects the intensity of spontaneous brain activity (Zang et al. 2007). fALFF measures the ratio of the power spectrum of low frequency to the whole detectable frequency range (Zou et al. 2008). The fALFF in the slow‐4 and slow‐5 bands is more sensitive and specific to reveal features of spontaneous brain activity (Yu et al. 2014). ReHo reveals the regional synchronization of spontaneous neural activity (Tononi et al. 1998; Zang et al. 2004). Unlike the FC method, these three parameters do not require seed selection, and they allow for the evaluation of abnormalities in certain brain regions at the whole‐brain level (Bu et al. 2019). In recent years, machine learning (ML) methods have been widely applied in neuroimaging data analysis and can extract effective information from rs‐fMRI data to aid early diagnosis and prognostic evaluation (Khosla et al. 2019; Pereira et al. 2009). Compared with traditional statistical analysis, ML is highly sensitive to spatially distributed information and could capture the nonlinear relationships among multiple features (Islam et al. 2022; Kyriazos and Poga 2024; Levy et al. 2020). This approach can identify features with diagnostic value for distinguishing patients from healthy controls (HCs) at the individual level and quantify the diagnostic efficacy of these features. A related study demonstrated that using ALFF and fALFF as features in combination with support vector machine (SVM) models can effectively differentiate patients with comitant exotropia (CE) from HCs (Chen et al. 2021). The study found that ALFF and fALFF could be sensitive biomarkers for distinguishing CE patients from HCs. In addition to SVM, other classifiers also show great potential in neuroimaging data classification (Pereira et al. 2009). To the best of our knowledge, the performance of various ML methods based on rs‐fMRI parameters in discriminating IXT children from HCs is unknown.

In the current study, we employed various ML methods to investigate the performance of different rs‐fMRI parameters within brain regions in distinguishing IXT children from HCs. Our study aimed to (i) investigate which rs‐fMRI parameters could effectively distinguish IXT children from HCs; (ii) select the best ML models of different rs‐fMRI parameters in identifying IXT children; and (iii) identify the brain regions with discriminative power in distinguishing IXT from HCs.

## 2 Materials and Methods

**FIGURE 1:**
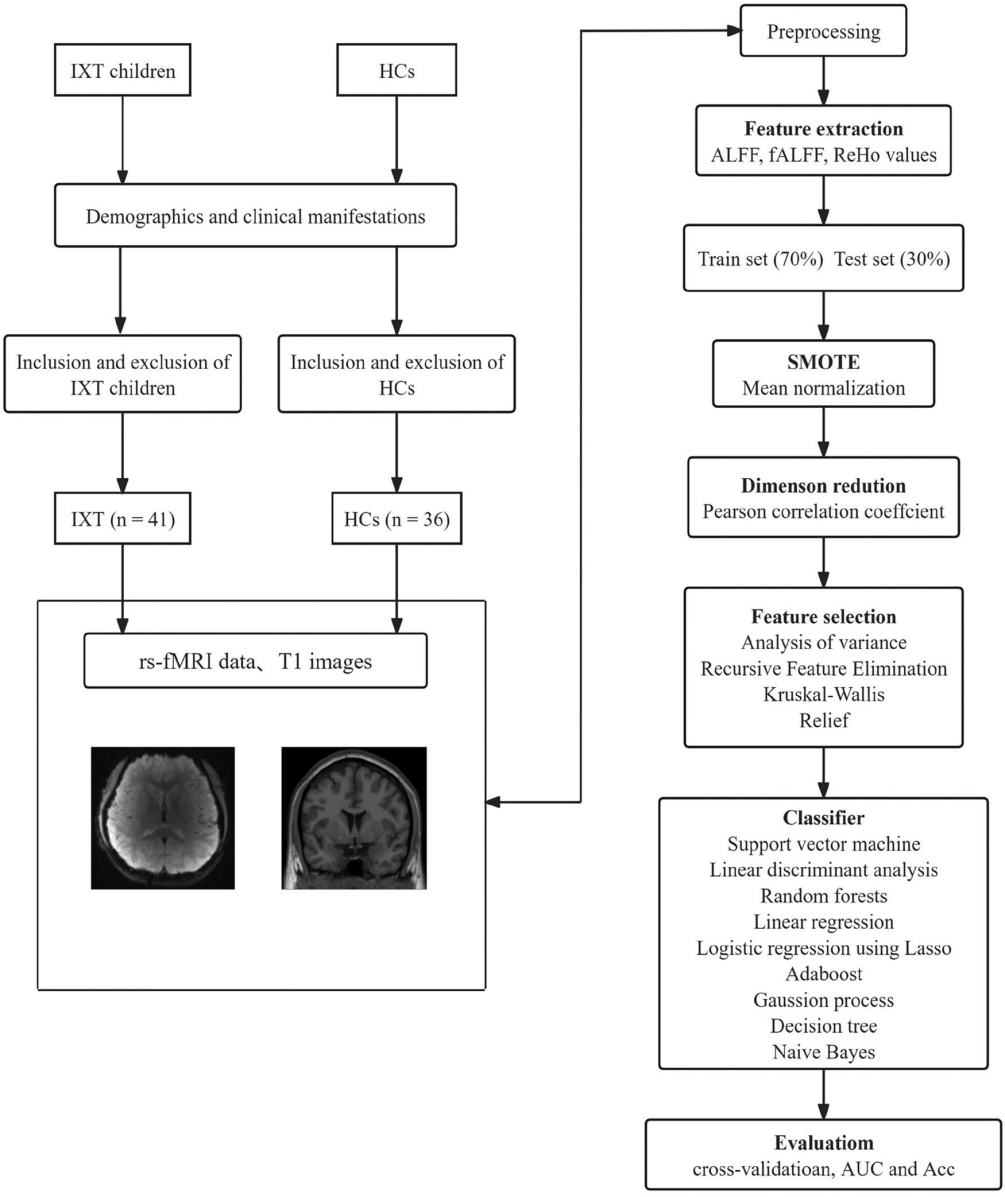
A schematic diagram of the MRI data collection, data processing, and machine learning analysis for the identification of IXT children. Abbreviations: rs‐fMRI, resting‐state functional magnetic resonance imaging; IXT, intermittent exotropia; HCs, healthy controls; AUC, area under the receiver operator characteristic curve; Acc, accuracy.

The pipeline of this study, involving MRI data collection, data processing, and ML analysis, is presented in Figure 1.

### 2.1 Participants

This study was approved by the medical research ethics committee and institutional review board of Capital Medical University, Beijing Tongren Hospital (No. TRECKY2020‐139), and written informed consent was obtained from all participants and their guardians. Forty‐one children with IXT and 36 age‐, sex‐, handedness‐, and education‐matched HCs were recruited.

The inclusion criteria for IXT children were as follows: (1) age between 9 and 16 years old; (2) diagnosed as IXT based on history and ocular examinations; (3) best corrected visual acuity (BCVA) ≥ 1.0. The exclusion criteria were (1) ocular disease; (2) history of eye surgery; (3) history of brain, psychiatric, or systemic disorders; (4) MR imaging contraindications (metal in cardiac pacemakers or prostheses); and (5) low‐quality MRI images.

### 2.2 MRI Data Acquisition

The participants were examined using a 3.0 T MRI scanner (Siemens Healthineers, Erlangen, Germany) with a standard 64 eight‐channel head coil. Foam pads were placed around the head to restrict head motion, and earplugs were used to reduce scanner noise.

The rs‐fMRI data were acquired using a simultaneous multi‐slice echo planar imaging sequence with the following parameters: time of repetition (TR) = 1500 ms, time of echo (TE) = 30 ms, flip angle = 70°, voxel size = 2.0×2.0×2.0 mm, field of view = 220 mm × 220 mm, matrix = 110×110, slice thickness = 2 mm, and 340 time points. The scanning time was 8 min and 45 seconds.

Three‐dimensional T1‐weighted anatomical images were obtained using a 3D magnetization prepared rapid acquisition gradient echo sequence: TR = 2000 ms, TE = 2.25 ms, inversion time (TI) = 900 ms, flip angle = 8°, voxel size = 1 × 1 × 1 mm, field of view = 256 mm×256 mm, matrix = 256 × 256, slice thickness = 1.0 mm, and 192 slices. The scanning time was 4 min and 6 seconds.

### 2.3 Resting‐State Data Preprocessing

The processing of image data was carried out using the DPABI (version 7.0; http://rfmri.org/dpabi/) (Yan et al. 2016) and SPM12 (Friston et al. 2007) toolboxes in MATLAB R2019b (The MathWorks, Natick, United States). The first 10 volumes were trimmed off during magnetization equilibration. The remaining images underwent slice timing and head motion correction. The data were then normalized into the standard space of the Montreal Neurological Institute with resampling to 3 mm isotropic voxels, and smoothed with a Gaussian kernel of 6 mm full‐width at half‐maximum (FWHM). Friston‐24 motion parameters, whole brain, white matter, and cerebrospinal fluid signals were regressed out as the covariates (Friston et al. 1996). Participant data with maximum head translation (rotation) motion > 4 mm (4°) were discarded.

### 2.4 Calculation of ALFF, fALFF, and ReHo

DPABI was used to calculate the zALFF, zfALFF, and zReHo values. The fast Fourier transformation converted the time series of each voxel to a frequency domain. The square root averaged across 0.01‐0.08 Hz at each voxel was taken as the ALFF value. The z‐score was calculated by subtracting the original ALFF value of the overall mean voxel from the whole voxel and then dividing by the standard deviation (SD) of the ALFF value of all voxels. This process was used to further remove individual differences in physiological noise interference and mean signal intensity according to the following formula: zALFF = (ALFF‐mALFF) / SD. The fALFF is a ratio of ALFF in a given frequency band to the ALFF over the entire detectable frequency range. The fALFF values at the slow‐4 band (0.027‐0.073 Hz) and the slow‐5 band (0.01‐0.027 Hz) were calculated. Then the fALFF values were normalized by z‐score transformation to obtain the zfALFF.

The similarity of the time series of a given voxel to those of its nearest 26 neighboring voxels is calculated using Kendall's coefficient of concordance (KCC). Individual ReHo values were generated by computing the KCC for each voxel across the entire brain. Then, the ReHo values were spatially smoothed (FWHM = 6 mm) and normalized by z‐score transformation to obtain the zReHo.

### 2.5 Feature Extraction

For each rs‐fMRI parameter (ALFF, slow‐4 fALFF, slow‐5 fALFF, and ReHo) of each participant, we calculated the mean zALFF, zfALFF, and zReHo values of each region of interest (ROI) using the homemade MATLAB codes. The 360 cortical areas of the Human Connectome Project multimodal parcellation atlas (HCP‐MMP 1.0 atlas) (Glasser et al. 2016) were chosen as 360 ROIs. Each rs‐fMRI parameter value of one cortical area was taken as a feature for further ML analysis.

### 2.6 Feature Selections and Classification

FeAture Explorer software (FAE, v 0.4.1, (Song et al. 2020), https://github.com/salan668/FAE) was used for feature selections and classification. The datasets were randomly divided into a training set (70%) and a test set (30%). Firstly, the synthetic minority oversampling technique (SMOTE) was conducted to balance the training dataset. The dataset was normalized using mean normalization. Secondly, Pearson correlation coefficient (PCC) was performed to reduce dimension. Finally, we used four feature selection methods, including analysis of variance (ANOVA), recursive feature elimination (RFE), Kruskal‐Wallis (KW), and relief. The feature number range was set from 1 to 20. Nine classifiers were applied to test the classification performances, including the support vector machine (SVM), linear discriminant analysis (LDA), random forests (RF), linear regression (LR), logistic regression using Lasso (LRLasso), ada‐boost (AB), naive Bayes (NB), Gaussian process (GP), and decision tree (DT). The parameter settings of the classification algorithms are presented in Table 1.

**TABLE 1:** The parameters of the algorithms.

Algorithms	Parameters
SVM	C = 1.0, kernel = ‘rbf’, degree = 3, gamma = ‘scale’, coef0 = 0.0, shrinking = True, probability = False, tol = 0.001, cache_size = 200, class_weight = None, verbose = False, max_iter = —1, decision_function_shape = ‘ovr’, break_ties = False, random_state = None
LDA	Solver = ‘svd’, shrinkage = None, priors = None, n_components = None, store_covariance = False, tol = 0.0001
RF	n_estimators = 100, *, criterion = ‘gini’, max_depth = None, min_samples_split = 2, min_samples_leaf = 1, min_weight_fraction_leaf = 0.0, max_features = ‘auto’, max_leaf_nodes = None, min_impurity_decrease = 0.0, min_impurity_split = None, bootstrap = True, oob_score = False, n_jobs = None, random_state = None, verbose = 0, warm_start = False, class_weight = None, ccp_alpha = 0.0, max_samples = None
LR	penalty = ‘l2’, *, dual = False, tol = 0.0001, C = 1.0, fit_intercept = True, intercept_scaling = 1, class_weight = None, random_state = None, solver = ‘lbfgs’, max_iter = 100, multi_class = ‘auto’, verbose = 0, warm_start = False, n_jobs = None, l1_ratio = None
LRLasso	alpha = 1.0, *, fit_intercept = True, normalize = False, precompute = False, copy_X = True, max_iter = 1000, tol = 0.0001, warm_start = False, positive = False, random_state = None, selection = ‘cyclic’
AB	base_estimator = None, *, n_estimators = 50, learning_rate = 1.0, algorithm = ‘SAMME.R’, random_state = None
GP	kernel = None, *, optimizer = ‘fmin_l_bfgs_b’, n_restarts_optimizer = 0, max_iter_predict = 100, warm_start = False, copy_X_train = True, random_state = None, multi_class = ‘one_vs_rest’, n_jobs = None
NB	alpha = 1.0, binarize = 0.0, fit_prior = True, class_prior = None
DT	criterion = ‘gini’, splitter = ‘best’, max_depth = None, min_samples_split = 2, min_samples_leaf = 1, min_weight_fraction_leaf = 0.0, max_features = None, random_state = None, max_leaf_nodes = None, min_impurity_decrease = 0.0, min_impurity_split = None, class_weight = None, ccp_alpha = 0.0

### 2.7 Evaluations

We evaluated the performance of ML models by standard performance metrics including area under the receiver operator characteristic curve (AUC), accuracy (Acc), sensitivity (Sen), specificity (Spe), positive predictive value (PPV), and negative predictive value (NPV) in ten‐fold cross‐validation.

### 2.8 Statistical Analysis

The SPSS 26.0 software package (SPSS, Inc., Chicago, IL, USA) was used for statistical analysis. The normality of demographic and clinical data was assessed using the Shapiro‐Wilk test. For non‐normally distributed data, such as ages, education, and the BCVA, we used the median (range) to report them. The Mann‐Whitney U test was used to compare non‐normally distributed data between groups. Categorical variables were conveyed as counts, and the comparison of genders was conducted using the Chi‐square test. The significance threshold was set at p < 0.05.

## 3 Results

### 3.1 Demographics and Clinical Manifestations

The demographics and clinical characteristics of 41 IXT children and 36 HC subjects are listed in Table 2. No significant differences were found between the two groups in age, gender, education, or the BCVA.

**TABLE 2:** Demographics and clinical characteristics of subjects included in the analysis.

	IXT (n = 41)	HC (n = 36)	p‐value
Gender (male/female)	19/22	19/17	0.650^a^
Age (years)	11.0 (9∼15)	11.0 (9∼16)	0.897^b^
Handedness (R/L)	41/0	36/0	—
Education (years)	5.0 (3∼9)	5.0 (3∼10)	0.975^b^
The best‐corrected VA (R)	1.0 (1.0∼1.2)	1.0 (1.0∼1.2)	0.491^b^
The best‐corrected VA (L)	1.0 (1.0∼1.2)	1.0 (1.0∼1.5)	0.440^b^

*Notes*: Data are presented as median (range) for continuous variables and number for categorical variables.

*Abbreviations*: HC, healthy control; IXT, intermittent exotropia; R, right; L, left; VA, visual acuity

^a^ The p‐value was assessed by the Chi‐squared test.

^b^ The p‐value was assessed by the Mann‐Whitney U test.

### 3.2 Performance of the ML Models Based on ALFF, fALFF and ReHo

The criteria for screening ML models were as follows: AUC of training/validation/test datasets ≥ 0.75, Acc of cross‐validation ≥ 0.75, and features < 10. The selected models were ranked by the AUC of the validation dataset generated from FAE software.

**FIGURE 2:**
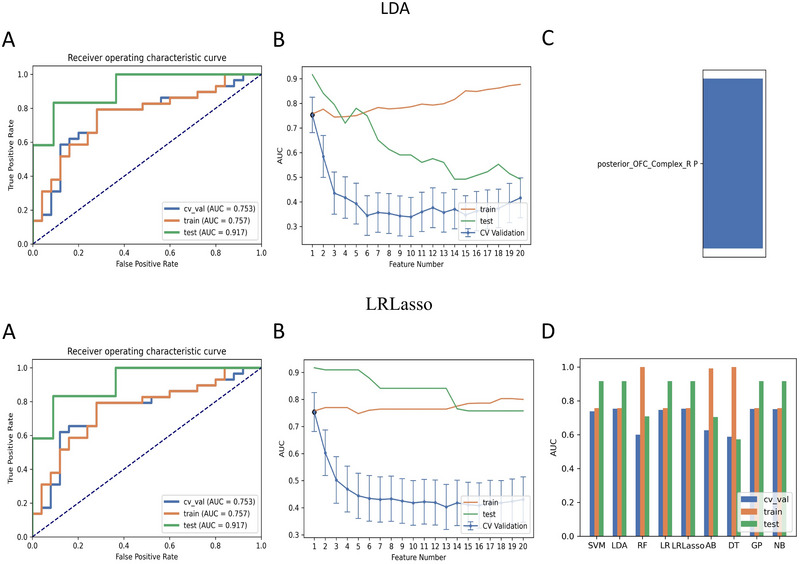
Two ML classifiers (linear discriminant analysis [LDA] and logistic regression using Lasso [LRLasso]) combined with Kruskal‐Wallis (KW) feature selection based on ALFF showed better performance among the selected models. (A) Receiver operating characteristic (ROC) curves of those models on different datasets, (B) FeAture Explorer software suggested a candidate 1‐feature model according to the “one‐standard error” rule, (C) The contribution of one feature in the two final models, and (D) Areas under the curve (AUCs) of different datasets using nine classifiers combined with KW feature selection. SVM, support vector machine; RF, random forests; LR, linear regression; AB, ada‐boost; DT, decision tree; GP, Gaussian process; NB, naive Bayes.

For the ALFF parameter, four ML classifiers with KW feature selection using one feature were selected, as shown in Table 3. The special feature was the mean zALFF on Posterior_OFC_Complex_R. Using KW feature selection, the LDA and LRLasso classifiers yielded the same AUC, which was the highest among all four ML models (Figure 2). The AUCs of the training, validation, and test datasets were 0.757, 0.753, and 0.917, respectively, and the Acc, Sen, and Spe values were 0.759, 0.793, and 0.720, respectively, for the LDA and LRLasso classifiers with KW feature selection.

**TABLE 3:** The ML models with AUC ≥ 0.75, Acc of cross‐validation ≥ 0.75, and features < 10 based on ALFF.

models name		AUC	Acc	Sen	Spe	PPV	NPV
	cross‐validation	0.753	0.759	0.793	0.720	0.767	0.750
Mean_PCC_KW_1_LDA	test	0.917	0.870	0.833	0.909	0.909	0.833
	train	0.757	0.759	0.793	0.720	0.677	0.750
	cross‐validation	0.753	0.759	0.793	0.720	0.767	0.750
Mean_PCC_KW_1_LRLasso	test	0.917	0.870	0.833	0.909	0.909	0.833
	train	0.757	0.759	0.793	0.720	0.677	0.750
	cross‐validation	0.752	0.759	0.793	0.720	0.767	0.750
Mean_PCC_KW_1_GP	test	0.917	0.870	0.833	0.909	0.909	0.833
	train	0.757	0.759	0.793	0.720	0.677	0.750
	cross‐validation	0.750	0.759	0.793	0.720	0.767	0.750
Mean_PCC_KW_1_NB	test	0.917	0.870	0.833	0.909	0.909	0.833
	train	0.757	0.759	0.793	0.720	0.677	0.750

Abbreviations: Acc, accuracy; AUC, area under the receiver operator characteristic curve; GP, Gaussian process; KW, Kruskal‐Wallis; LDA, linear discriminant analysis; LRLasso, logistic regression using Lasso; NB, naive Bayes; NPV, negative predictive value; PCC, Pearson correlation coefficient; PPV, positive predictive value; Sen, sensitivity; Spe, specificity.

**FIGURE 3:**
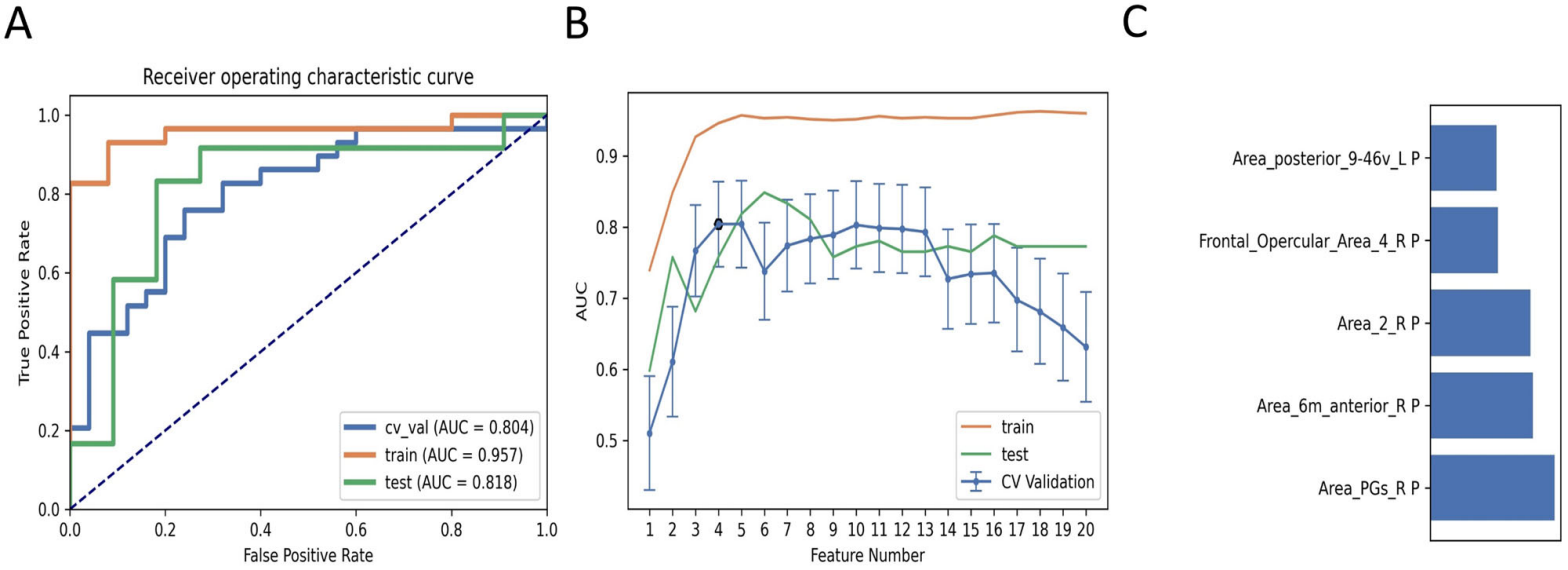
The linear regression (LR) classifier and Analysis of Variance (ANOVA) feature selection based on slow‐5 fALFF showed the best performance among the selected models. (A) Receiver operating characteristic (ROC) curves of this model on different datasets, (B) FeAture Explorer software suggested a candidate 5‐feature model according to the “one‐standard error” rule, and (C) The contribution of features in the final model.

For the fALFF parameter, five models were selected based on slow‐5 fALFF, as shown in Table 4. Considering the limited space, the results of slow‐4 fALFF were not presented because the AUCs of the training, validation, and test datasets of the ML models based on the slow‐4 fALFF failed to reach 0.75. The ML model combining the LR classifier with ANOVA feature selection yielded the highest AUC using 5 features, including the mean slow‐5 zfALFF values of Area_PGs_R, Area_6m_anterior_R, Area_posterior_9‐46v_L, Area_2_R, and Frontal_Opercular_Area_4_R (Figure 3). The AUCs of the training, validation, and test datasets achieved 0.957, 0.804, and 0.818, respectively, and the Acc, Sen, and Spe values were 0.759, 0.759, and 0.760, respectively.

**TABLE 4:** The ML models with AUC ≥ 0.75, Acc of cross‐validation ≥ 0.75, and features < 10 based on slow‐5 fALFF.

models name		AUC	Acc	Sen	Spe	PPV	NPV
	cross‐validation	0.804	0.759	0.759	0.760	0.786	0.731
Mean_PCC_ANOVA_5_LR	test	0.818	0.826	0.833	0.818	0.833	0.818
	train	0.957	0.926	0.931	0.920	0.931	0.920
	cross‐validation	0.800	0.756	0.931	0.560	0.711	0.880
Mean_PCC_ANOVA_4_LDA	test	0.750	0.739	0.833	0.636	0.714	0.778
	train	0.946	0.907	0.862	0.960	0.962	0.857
	cross‐validation	0.796	0.759	0.793	0.720	0.767	0.750
Mean_PCC_KW_5_LR	test	0.765	0.739	0.583	0.909	0.875	0.667
	train	0.952	0.907	0.862	0.960	0.962	0.857
	cross‐validation	0.785	0.759	0.897	0.600	0.722	0.833
Mean_PCC_ANOVA_5_LDA	test	0.795	0.783	0.750	0.818	0.818	0.750
	train	0.952	0.907	0.828	1.000	1.000	0.833
	cross‐validation	0.766	0.778	0.931	0.600	0.730	0.880
Mean_PCC_ANOVA_5_SVM	test	0.811	0.783	0.667	0.909	0.889	0.714
	train	0.952	0.907	0.828	1.000	1.000	0.833

Abbreviations: Acc, accuracy; ANOVA, analysis of variance; AUC, area under the receiver operator characteristic curve; KW, Kruskal‐Wallis; LDA, linear discriminant analysis; LR, linear regression; NPV, negative predictive value; PCC, Pearson correlation coefficient; PPV, positive predictive value; Sen, sensitivity; Spe, specificity; SVM, support vector machine.

**FIGURE 4:**
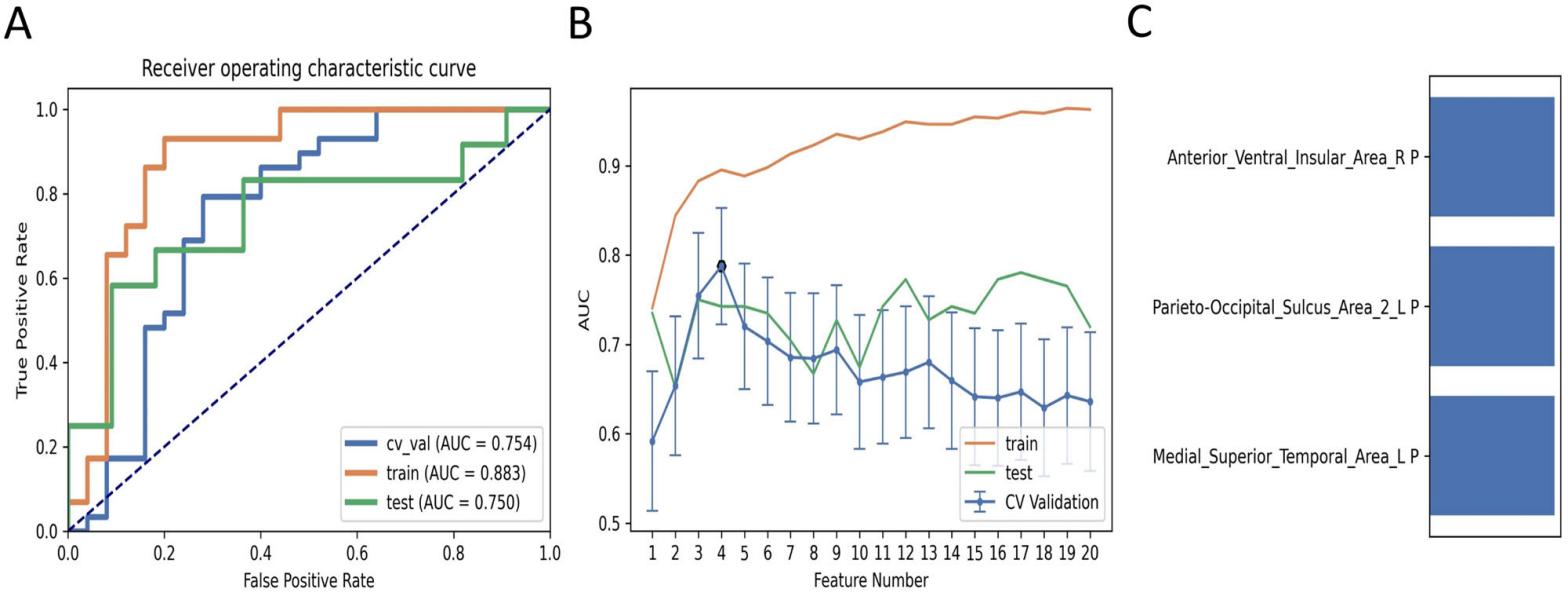
The Gaussian process (GP) classifier and Recursive Feature Elimination (RFE) feature selection based on ReHo showed the best performance among all the ML models. (A) Receiver operating characteristic (ROC) curves of this model on different datasets, (B) FeAture Explorer software suggested a candidate 3‐feature model according to the “one‐standard error” rule, and (C) The contribution of features in the final model.

Regarding the ReHo parameter, the ML model that combined the GP classifier with RFE feature selection obtained the highest AUC with three features (the mean zReHo values of Medial_Superior_Temporal_Area_L, Anterior_Ventral_Insular_Area_R, and Parieto‐Occipital_Sulcus_Area_2_L), as shown in Table 5 and Figure 4. The AUCs of the training, validation, and test datasets achieved 0.883, 0.754, and 0.750, respectively, and the Acc, Sen, and Spe values were 0.759, 0.793, and 0.720, respectively.

**TABLE 5:** The ML models with AUC ≥ 0.75, Acc of cross‐validation ≥ 0.75, and features < 10 based on ReHo values.

models name		AUC	Acc	Sen	Spe	PPV	NPV
	cross‐validation	0.754	0.759	0.793	0.720	0.767	0.750
Mean_PCC_RFE_3_GP	test	0.750	0.739	0.583	0.909	0.875	0.667
	train	0.883	0.870	0.931	0.800	0.844	0.909

Abbreviations: Acc, accuracy; AUC, area under the receiver operator characteristic curve; GP, Gaussian process; NPV, negative predictive value; PCC, Pearson correlation coefficient; PPV, positive predictive value; RFE, Recursive Feature Elimination; Sen, sensitivity; Spe, specificity.

### 3.3 Discriminative Brain Regions Between IXT Children and HCs Based on ALFF, Slow‐5 fALFF, and ReHo

**FIGURE 5:**
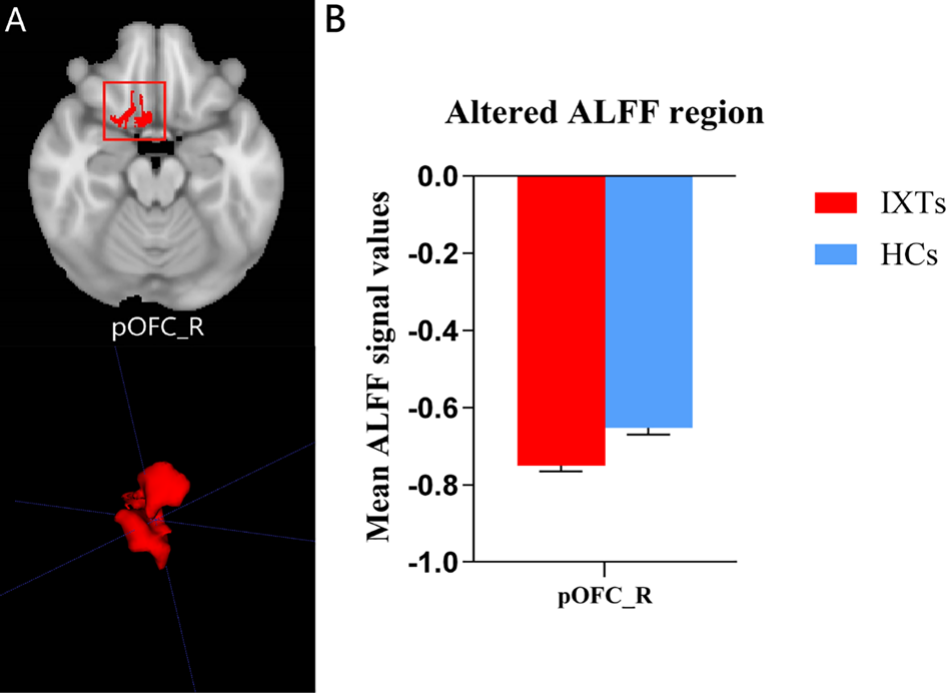
The discriminative brain region between IXT children and the HCs was identified based on the ALFF parameter. Decreased ALFF values in the HCP‐MMP‐right pOFC (posterior orbitofrontal cortex) were observed in IXT children. (A) The location and three‐dimensional (3D) display of the right pOFC on the brain. (B) The mean ALFF values of the identified brain region. Abbreviations: IXT, intermittent exotropia; HCs, healthy controls.

**FIGURE 6:**
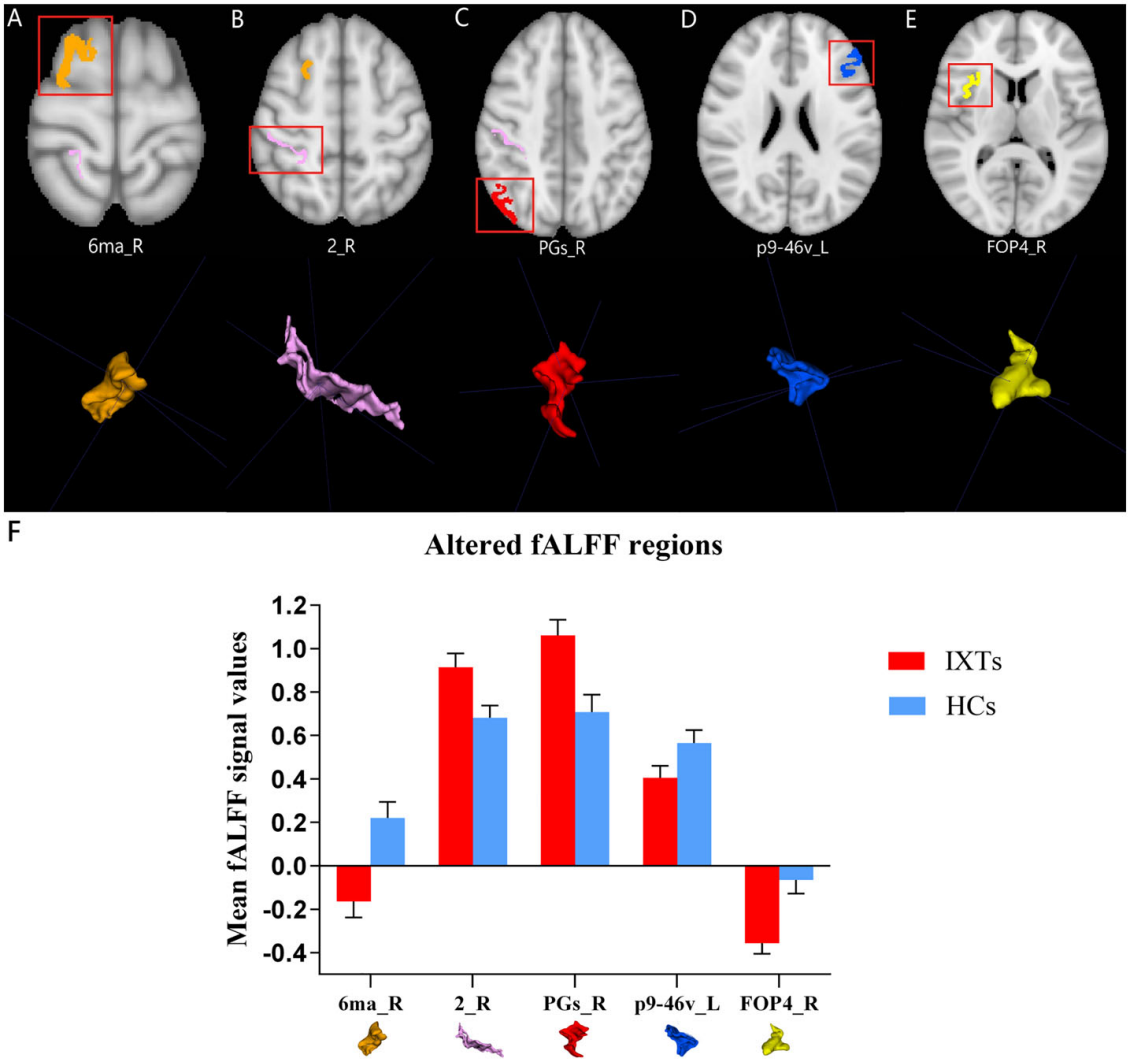
The discriminative brain regions between IXT children and the HCs were identified based on the slow‐5 fALFF parameter. Decreased slow‐5 fALFF values in the HCP‐MMP‐right 6ma (supplementary motor area), HCP‐MMP‐left p9‐46v (dorsolateral prefrontal cortex), and HCP‐MMP‐right FOP4 (frontal opercula), and increased slow‐5 fALFF values in the HCP‐MMP‐right area2 (primary somatosensory complex) and HCP‐MMP‐right PGs (inferior parietal gyrus) were observed in IXT children. (A‐E) The location and three‐dimensional (3D) display of right 6ma, area 2, PGs, left p9‐46v, and right FOP4 on the brain. (F) The mean slow‐5 fALFF values of the identified brain regions. Abbreviations: IXT, intermittent exotropia; HCs, healthy controls.

**FIGURE 7:**
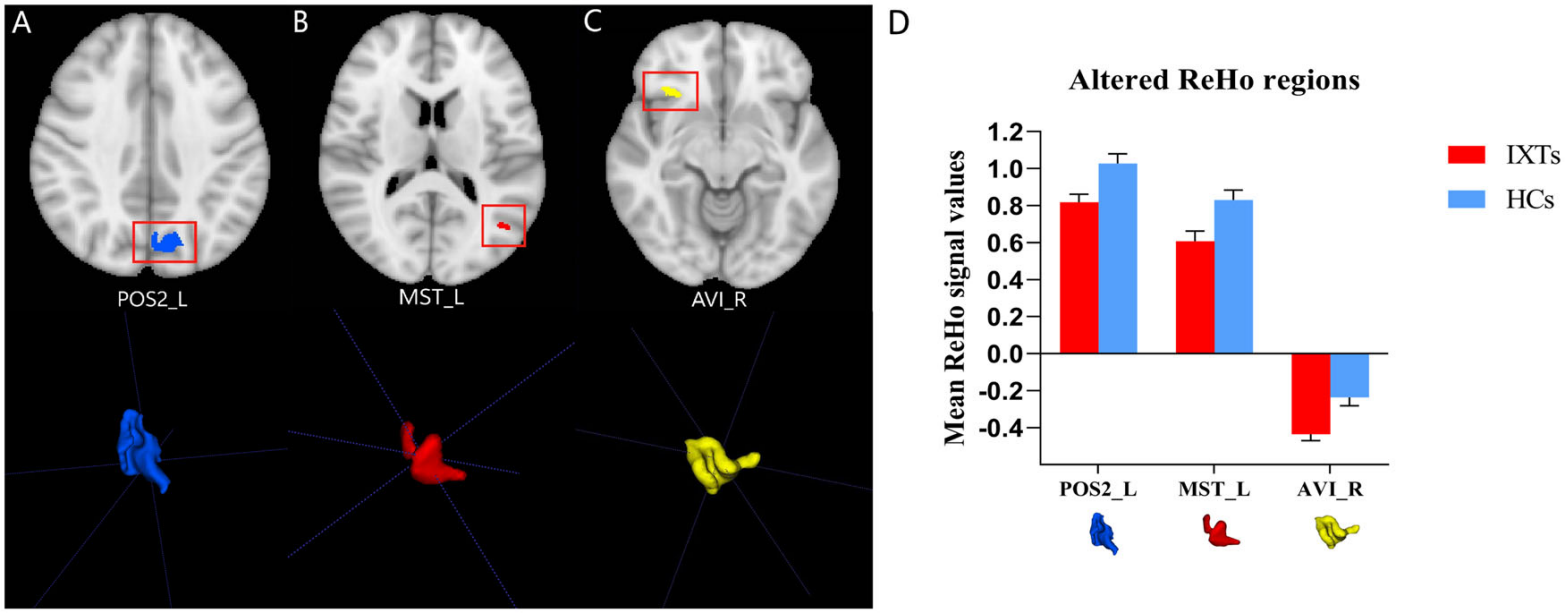
The discriminative brain regions between IXT children and the HCs were identified based on the ReHo parameter. Decreased ReHo values in the HCP‐MMP‐left MST (medial superior temporal, MST), HCP‐MMP‐right AVI (anterior ventral insula), and HCP‐MMP‐left POS2 (parieto‐occipital sulcus) were observed in IXT children. (A‐C) The location and three‐dimensional (3D) display of left POS2, MST, and right AVI. (D) The mean ReHo values of the identified brain regions. Abbreviations: IXT, intermittent exotropia; HCs, healthy controls.

The discriminative brain region between IXT children and the HCs identified in the ALFF map was HCP‐MMP‐right pOFC (posterior orbitofrontal cortex, pOFC), as shown in Figure 5. The brain regions that contributed to the identification of IXT children in the slow‐5 fALFF map included HCP‐MMP‐right PGs (inferior parietal gyrus, IPG) (Glasser et al. 2016), HCP‐MMP‐right 6ma (supplementary motor area, SMA), HCP‐MMP‐right area2 (primary somatosensory complex), HCP‐MMP‐right FOP4 (frontal opercula), and HCP‐MMP‐left p9‐46v (dorsolateral prefrontal cortex, DLPFC), as shown in Figure 6. The discriminative brain regions between IXT children and the HCs in the ReHo map included HCP‐MMP‐left MST (medial superior temporal, MST), HCP‐MMP‐right AVI (anterior ventral insula), and HCP‐MMP‐left POS2 (parieto‐occipital sulcus, POS), as shown in Figure 7.

## 4 Discussion

Our current study investigated the potential diagnostic performance of different rs‐fMRI parameters within brain regions in distinguishing IXT children from HCs, and found out which ML models were suitable for our application. The regional neural activity across the whole brain reflected by ALFF, slow‐5 fALFF, and ReHo could distinguish IXT children from HCs. Among these rs‐fMRI parameters, the slow‐5 fALFF parameter showed the best classification performance. Based on the slow‐5 fALFF parameter as the feature, LR with ANOVA was the best ML model for distinguishing between IXT children and HCs. In addition, brain areas related to stereopsis, eye movement, and high‐order cognitive functions contributed to the identification of IXT children.

Classification performance varied among different rs‐fMRI parameters. Of the four measurements, the slow‐5 fALFF parameter showed the best classification performance. Based on the slow‐5 fALFF values of five cortices (right IPG, right SMA, right primary somatosensory complex, right frontal opercula, and left DLPFC) as the input features, LR with ANOVA was the best ML model for discriminating IXT children from HCs, indicating the slow‐5 fALFF parameter can be a promising biomarker for distinguishing children with IXT from HCs. It is suggested that slow‐5 bands are more sensitive in reflecting the signal from cortical regions (Yu et al. 2014). Previous studies also supported our findings. Jia et al. found that the slow‐5 band showed more sensitivity in detecting the abnormal low‐frequency oscillation amplitude and successfully distinguished patients with tension‐type headache from HCs using slow‐5 fALFF as the feature (Jia et al. 2024). A study on hypothyroidism also observed greater fALFF abnormalities in the slow‐5 band than the standard band and slow‐4 band (Shi et al. 2024). For the ALFF parameter, four ML classifiers with KW feature selection based on the mean ALFF on right pOFC were selected, which indicates that the mean ALFF on right pOFC is a stable feature for distinguishing children with IXT from HCs. Regarding the ReHo parameter, the GP classifier with RFE feature selection had better performance in distinguishing between IXT children and HCs compared with other ML models. In general, for the identification of IXT children, the optimal ML models (in terms of the classifiers and feature selection methods) varied with different rs‐fMRI parameters. Based on the slow‐5 fALFF, the best ML model was LR with ANOVA. For the ALFF, the best ML model was LDA and LRLasso with KW. Recording the ReHo, the best ML model was GP with RFE. In the study of discriminating IXT children from HCs, it is important to apply a variety of ML methods rather than relying on a single method.

Our finding revealed that brain areas associated with stereopsis and eye movement contributed to the discrimination of IXT children from HCs. Decreased ReHo in the left POS and MST, decreased fALFF in the right SMA, and increased fALFF in the right IPG and primary somatosensory complex were observed in IXT children. The POS serves as a neural interface between the dorsal and ventral visual streams in the process of disparity‐defined near and far space (Wang et al. 2016; Wang et al. 2019). The MST is involved in the execution of smooth pursuit eye movements (Ilg 2008). A previous study reported CS children showed decreased cortical thickness in the POS (Yin et al. 2021). The SMA participates in the saccadic task and controls eye movement (Campos et al. 2005; Chen et al. 2021; O'Driscoll et al. 1995). Alterations of the POS, MST, and SMA may lead to impaired advanced cortical processing of visual information and oculomotor disorders. As part of the motor and sensory network, the primary somatosensory complex receives general bodily sensation and connects with the primary motor complex for precise execution of eye movement (Lee et al. 2013). The IPG is a key region in the dorsal visual stream, and the pathway is primarily engaged in eye movement and spatial position information processing (Barany et al. 2020; Rizzolatti and Matelli 2003). Consistent with our finding, He et al. found increased ALFF/fALFF in the right IPG of IXT adults (He et al. 2021). In IXT children, part of the stereovision function and eye position control ability still exist before developing into constant exotropia. Consequently, the increased fALFF in the right IPG and primary somatosensory complex might reflect functional compensation.

The discriminative brain areas identified in our study also included regions associated with higher‐order cognitive functions. Decreased fALFF and ALFF in the left DLPFC, right pOFC, anterior ventral insula, and frontal opercula were found in IXT children. The prefrontal cortex (PFC), which encompasses DLPFC and OFC, is a collection of cortical regions involved in cognitive functions (Yeterian et al. 2012). Furthermore, the PFC interconnects with visual areas and modulates top‐down attention control (Clark et al. 2015). The DLPFC is important for cognitive control, spatial attention, and spatial working memory (Clark et al. 2015; Funahashi et al. 1989; Funahashi et al. 1993). The OFC is essential in the process of visuo‐affective prediction (Chaumon et al. 2014). The insula and opercula function as a multimodal sensory center, responsible for cognitive memory, self‐motion perception, and eye movements (Dieterich et al. 1998; Nagai et al. 2007). Abnormal cortical thickness and ALFF in the anterior insula, OFC, and PFC were also reported in CS children (Yin et al. 2021). Combined with the previous study, we speculate that dysfunction in high‐order cortical regions may cause the disruption of the top‐down visual attention modulation system in IXT children.

We acknowledge that our research has several limitations. First, this was a small sample study, and future studies with larger sample sizes might provide deeper insights. Second, due to the lack of clinical characteristics in a small number of patients, correlations between rs‐fMRI parameter values in identified brain regions and clinical features were not analyzed. Third, children under 9 years old were not recruited because they could not cooperate well during MRI examinations, so data from these early years are lacking.

## 5 Conclusion

In conclusion, our study demonstrated that ML methods combined with rs‐fMRI parameters could effectively distinguish IXT children from HCs, with the slow‐5 fALFF providing the best classification performance. Based on the slow‐5 fALFF parameter as the feature, LR with ANOVA was the best ML model for distinguishing between IXT children and HCs. The slow‐5 fALFF parameter could be a promising biomarker for identifying IXT children. The ML models varied with different rs‐fMRI parameters, highlighting the importance of applying multiple ML methods rather than relying on a single ML method when studying the classification of IXT children. Additionally, our findings emphasized the role of brain areas associated with stereopsis, eye movement, and higher‐order cognitive functions in the neuropathologic mechanisms underlying IXT children.

## Author Contributions

**Mengdi Zhou**: conceptualization, data curation, formal analysis, writing–original draft, visualization. **Huixin Li**: data curation, formal analysis, validation. **Xiaoxia Qu**: conceptualization, software, writing–review and editing. **Lirong Zhang**: data curation. **Xueying He**: data curation. **Xiwen Wang**: validation. **Jie Hong**: resources. **Jing Fu**: resources, funding acquisition. **Zhaohui Liu**: resources, project administration, supervision.

## Ethics Statement

The study was conducted in accordance with the Declaration of Helsinki and approved by the Ethics Committee and Institutional Review Board of Capital Medical University, Beijing Tongren Hospital (TRECKY2020‐139).

## Consent

The participants and their guardians provided their written informed consent to participate in this study.

## Conflicts of Interest

The authors declare no conflicts of interest.

### Peer Review

The peer review history for this article is available at https://publons.com/publon/10.1002/brb3.70556

## Data Availability

The data that support the findings of this study are available on request from the corresponding author. The data are not publicly available due to privacy or ethical restrictions.
